# KeratoEL: Detection of keratoconus using corneal parameters with ensemble learning

**DOI:** 10.1002/hsr2.2202

**Published:** 2024-06-30

**Authors:** Prodeep Kumar Paul, Arif Hossan, Shah Muhammad A. Ullah

**Affiliations:** ^1^ Department of Electronics and Communication Engineering Khulna University of Engineering & Technology (KUET) Khulna Bangladesh

**Keywords:** artificial neural network, decision tree, ensemble learning, keratoconus, support vector machine

## Abstract

**Background and Aims:**

Keratoconus is a progressive eye condition in which the normally round cornea thins and bulges outwards into a cone shape. This irregular shape causes light to scatter in multiple directions as it enters the eye, leading to distorted vision, increased sensitivity to light and frequent changes in the prescription of glasses or contact lenses. Detecting keratoconus at an early stage is not only difficult but also challenging.

**Methods:**

The study has proposed an ensemble‐based machine learning (ML) technique named KeratoEL to detect keratoconus at an early stage. The proposed KeratoEL model combines the basic machine learning algorithms, namely support vector machine (SVM), decision tree (DT), random forest (RF) and artificial neural network (ANN). Before employing the ML model for keratoconus detection, the data set is first preprocessed manually by eliminating some features that don't contribute any significant value to predict the exact class. Moreover, the output features are labelled into three different classes and Extra Trees Classifier is used to find out the important features. Then, the features are sorted in descending order and top 45, 30, and 15 features are taken as input datasets against the output. Finally, different machine learning models are tested using the input datasets and performance metrics are measured

**Results:**

The proposed model obtains 98.0%, 98.9% and 99.8% accuracy for top 45, 30, and 15 number of features respectively. Overall experimental results show that the proposed ensemble model outperforms the existing machine learning models.

**Conclusion:**

The proposed KeratoEL model effectively detects keratoconus at an early stage by combining SVM, DT, RF, and ANN algorithms, demonstrating superior performance over existing models. These results underscore the potential of the KeratoEL ensemble approach in enhancing early detection and treatment of keratoconus.

## 1 INTRODUCTION

Keratoconus is a corneal disorder marked by gradual thinning, leading to the protrusion of the cornea and a decline in visual acuity, without an inflammatory component.^1^ Typically, keratoconus manifests through progressively worsening myopia and astigmatism, resulting in suboptimal vision correction with glasses, challenges in adapting to contact lenses, visual exhaustion, and frequent headaches. The occurrence of keratoconus can vary, with a prevalence ranging from 0.2 to 4.790 cases per 100,000 individuals.^2^ Keratoconus may impact a single eye or both eyes simultaneously, exhibiting varying degrees of progression. Repetitive eye rubbing is widely recognized as the primary contributing factor to the progress of this disease.^3^ Keratoconus is typically diagnosed through the evaluation of corneal topography and the analysis of specific biomechanical properties of the cornea.^4^

The symptoms of keratoconus may vary depending on the stage of the disease. Considering that keratoconus typically manifests during puberty, primarily affecting children, the development and design of novel diagnostic tools for early disease detection could play a pivotal role in preventing or slowing down its progression.^5^ This, in turn, could safeguard the vision of young individuals. When the symptoms become obvious, an ophthalmologist can easily diagnose the keratoconus. Nevertheless, diagnosing suspect cases or those in the initial stages of the disease can be challenging, as symptoms may not be obvious and necessitate a more thorough examination of corneal features such as topography, elevations, thickness, and biomechanical qualities etc. There are multiple techniques that have been proposed to identify keratoconus eyes using corneal topography data. The majority of methods, however, depend on a subjective interpretation of topographical maps, which is prone to observer bias.^6^

Typically, keratoconus diagnosis involves a manual assessment conducted by specialists. These experts carefully evaluate various corneal attributes to gather the necessary data for confirming the presence of keratoconus. Nevertheless, to enhance specialist support in the diagnosis of keratoconus, numerous researchers have embraced machine learning (ML) algorithms to reinforce ophthalmologists' assessments concerning the presence of keratoconus in patients.^7^ The coordination of the experienced specialists along with the capabilities of ML in processing diverse data types holds the promise of reliably and accurately detecting keratoconus, even in its subclinical stage.^7^ This advancement not only expands the treatment options available to patients but also reduces the necessity for surgical interventions. Despite numerous technological advancements, accurately detecting early‐stage keratoconus remains a challenging endeavor.

ML algorithms have gained popularity over the years due to their wide‐ranging advantages for keratoconus detection and classification. However, various ML algorithms come with their unique strengths and limitations. So, building a hybrid ML model by integrating several models and utilizing their advantages allows for a superior model to be built. Herein, the primary objective is to utilize the benefits while eliminating the constraints associated with any particular model. Therefore, this paper proposes a hybrid ML model, known as ensemble learning, for early‐stage keratoconus detection and classification. The major contributions of this work are summarized as below

i) This study is focused on creating an efficient hybrid machine learning model for the early detection and classification of keratoconus, a critical step in improving patient outcomes. The proposed model, KeratoEL, combines SVM, DT, RF and ANN in the averaging ensemble method.
ii) The high dimensional data has been reduced into the most optimal feature set by employing the Extra Trees Classifier. As a result, the operational model exhibits improved performance without added processing complexity.
iii) The optimal feature set is categorized into three distinct subsets, comprising the top 45, 30, and 15 features, respectively. A thorough analysis has been conducted to assess how the number of features influences the model's performance across a range of feature sets, thereby validating its overall impact.
iv) The model's performance is rigorously assessed by examining its effectiveness on both standardized and normal data using various feature sets to see the valuable insights into how the model makes its decisions.
v) The study evaluates the effectiveness of the proposed ensemble model and compares it with state‐of‐the‐art models. The results demonstrate the model's ability to generalize and its accuracy in making predictions.

The remaining section of this paper is organized as follows. Section 2 provides a comprehensive literature review of the work. Section 3 includes an in‐depth overview of our proposed approach, including pre‐processing unit and classification model. Sections 4 and 5 present a brief description of the basic machine learning model and the evaluation metrics employed for the analysis of our proposed model, respectively. Section 8 includes experimental setup and the overall performance of our proposed model has been narrated in Section 7. Finally, Section 8 draws the conclusions of our work.

## 2 RELATED WORKS

The noninflammatory keratoconus frequently results in irreversible vision loss when it advances into the fourth decade of life. Considering the importance of its early onset, there is a growing need to develop tools and diagnostic techniques to detect keratoconus early, especially in the younger population, to stop or slow its progression. ML has become more popular in keratoconus detection. ML aided keratoconus detection is reliable and unbiased, which is more important in cases of early detection.^8^

Four sets of classifiers, including the multilayer perceptron (MLP),^9^ radial basis function network (RBFN),^10^ neural network (NN), and support vector machine (SVM)^11^ have been tested in keratoconus diagnosis. Among the four classifiers, the MLP achieves the highest accuracy of 92.2%, while the SVM has the lowest accuracy, which is 84.42%. A learning system based on a neural network that enables the diagnosis of keratoconus is presented in.^12^ The obtained results show that the test data set has the best accuracy, achieving 97.33%. However, the algorithm uses an excessive number of parameters, which makes it challenging to train and test. In,^13^ binary decision tree (DT) is applied for keratoconus diagnosis from corneal topography data and obtain an accuracy of 95%. The drawback of the proposed scheme is the small data set. In,^14^ the authors suggested a categorization method employing corneal shape data from optical coherence tomography (OCT) based devices and achieved 92% accuracy with 244 eyes. Nonetheless, the study lacks details regarding the severity level of keratoconus in the eyes and whether it included cases at the early stages of the condition.

An automated keratoconus diagnosis technique utilizing artificial intelligence (AI) is presented in^15^ with 91% accuracy. The algorithm utilizes a collection of topography images collected using a Pentacam^16^ that have been classified into two categories by experts (keratoconus eyes and non‐keratoconus eyes). The drawback of the proposed method is that the training data set comprises only 82 images. The authors in^17^ used the SVM method to distinguish between people with keratoconus and others who are healthy. This study utilized 860 eyes with pentacam data, which were divided into five distinct groups: 454 eyes with keratoconus, 67 eyes with forme fruste, 28 eyes with astigmatism, 117 eyes following refractive surgery, and 194 normal eyes. This method analyzes 22 factors with an accuracy of 98.9%, 93.1% and 88.8% for three different classification tasks (keratoconus vs. normal eyes, forme fruste vs. normal eyes and all 5 groups). For identifying patients with early‐stage keratoconus, a logistic regression statistical model was applied in.^18^ The sensitivity and specificity values in the application group were 85.0% and 86.7%, respectively. However, the model is applied only to young patients, overlooking its diagnostic applicability in older patients.

In,^14^ SVM classifier based model is developed for disease detection using corneal measurements through the integration of a Scheimpflug camera and Placido corneal topography. The classifier exhibited outstanding accuracy, whether or not it incorporated data generated from the posterior corneal surface and corneal thickness. In both scenarios, the accuracy exceeded 95%. Furthermore, when incorporating both anterior and posterior corneal data, SVM achieved an improved accuracy of over 97% for normal eyes. The keratoconus diagnosis is aided by the use of a convolutional neural network (CNN) in the KeratoDetect algorithm.^19^ The algorithm has been reported to show 99.33% accuracy, establishing it as a reliable screening tool for ophthalmologists. Since collecting real eye topographies is difficult, the data set is generated by the SyntEye KTC model. However, the algorithm's assessment does not consider this to be a bias. The authors in^5^ developed a ML algorithm for keratoconus diagnosis based on corneal imaging data. A number of ML algorithms were applied and tested against real medical data, specifically elevation, corneal topography and corneal pachymetry etc. obtained from OCT based corneal topography. The accuracy of 25 different applied models varies from 62% to 94% and cubic SVM achieved the highest accuracy of 94%. In,^20^ Random Forest (RF) has been utilized for classification among normal, suspect irregular and keratoconic corneas. The highest accuracy achieved in this study is 91.5%. However, the model relies on the classification of images into their respective groups. In,^21^ CNN is applied for normal and keratoconic eye detection based on the axial map of the anterior corneal surface. The model utilizes the images of 3000 normal eyes and 3000 keratoconic eyes collected by a pentacam. The model is able to achieve a maximum accuracy of 99.5%. However, this study didn't consider the Suspect Keratoconus case during the evaluation.

From the literature review discussed so far, it is clear that there is significant interest and ongoing research in the area of keratoconus diagnosis using a variety of methods, including statistical models, ML, and AI. Although several studies have shown promising results, there is still a need to improve the accuracy and precision of these techniques and to address the issue of false positive detection. The potential of developing a hybrid model that combines discrete learning models and utilizes the benefits of each model remains underdeveloped in this domain. Moreover, to utilize the advantages and reduce the limitations of discrete models, the decision has been made to develop an efficient hybrid model by combining multiple models. This hybrid model performs better in early disease detection and intervention than discrete models.

## 3 METHODOLOGY

**Figure 1:**
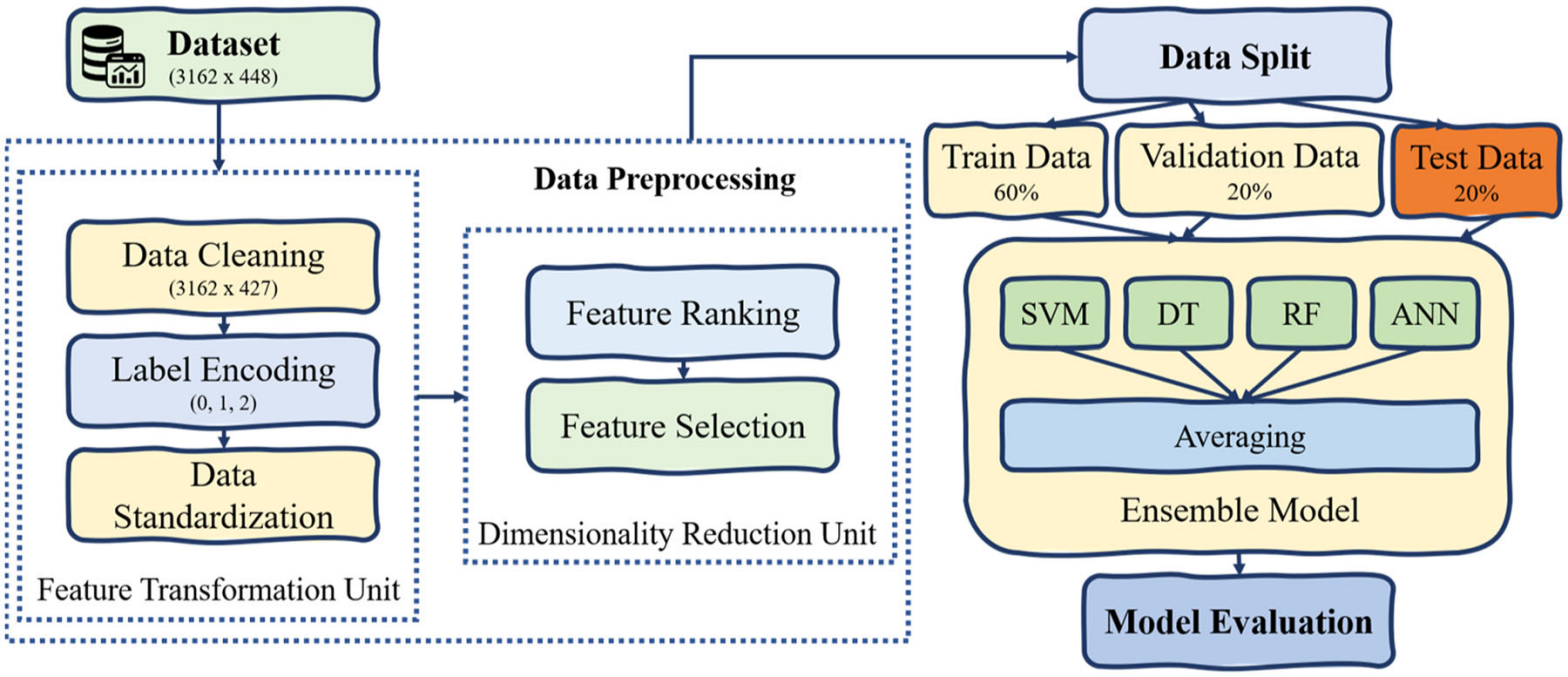
Proposed methodology with the ensemble technique.

In this paper, an ensemble‐based novel machine learning model, KeratoEL, is proposed for the detection of keratoconus. Our proposed model primarily comprises two key components: a pre‐processing unit and an ensemble model designed for the classification stage. Within the pre‐processing unit, the initial step involves the transformation of features, where each feature is first encoded into a label format at the input layer. The second step of the system incorporates a dimensionality reduction component designed to mitigate the challenges posed by the curse of dimensionality. For this feature reduction task, the Extra Trees Classifier has been employed as the foundation. The Extra Trees Classifier transforms the features of the original data set into a specific number of principal components, and only a subset of these components is chosen for the detection model. This process is consistent for the training, validation and testing datasets. Subsequently, a data standardization process is executed. Following the completion of the pre‐processing phase, the resulting reduced feature set is directed into the model. It is a heterogeneous ensemble technique that utilizes the advantages of four different classifiers, such as SVM, DT, RF and ANN. The operational flow diagram of the proposed model is shown in Figure 1.

### 3.1 Data description

Using the SS‐1000 CASIA OCT Imaging Methods (Tomey, Japan) and additional parameters from the electronic health record (EHR) system, we obtained corneal optical coherence tomography (OCT) pictures from 12,242 eyes of 3162 individuals. Without any prerequisites, every piece of data accessible at each apparatus was collected. Then, we chose a single visit from each eye while excluding any eyes that lacked an Ectasia Status Index (ESI). 3156 eyeballs in total satisfied the requirement. The average age of the participants was 69.7 (SD = 16.2) years, with almost 57% of the individuals being female.^22^ The ESI index of CASIA was used to create three screening labels: normal for values ranging between 0 and 4, forme fruste keratoconus (or keratoconus‐suspect) for values between 5 and 29, and keratoconus for values of 30 or higher. This data set, which used CASIA labels, includes 390 keratoconus cases, 796 forme‐fruste keratoconus cases, and 1970 healthy eyes. The Belin‐Ambrósio (BA) index and the instrument‐guided screening index (ESI) have been found to have good agreement in the diagnosis of keratoconus.^22^ So, the data set, which we collected, has a size of 3162 records with 448 features. Among them, one last column is the target column to which our trained model will be mapped.

### 3.2 Data cleaning

We had to drop several columns before training models. Because some of them include a constant number. Some of them include null values. Some of them include the same string. But all of them are actually not contributing to training our machine learning models. So, in total, we dropped 21 parameters from the data set for cleaning purposes. In total, 21 parameters were dropped from the original data set and they are presented in Table 1.

**Table 1:** Dropped parameters and reason for dropping.

Parameter name	Reason for dropping
idEye	Not a Number
BFS_Ecc	All are zeros
BFS_Ecc.1	All are zeros
Apex.1	All are zeros
Thinnest.4 mm.	Constant value
LocationX.2	All are zeros
LocationY.2	All are zeros
CSI_T.1	All are zeros
SD_T.4 mm.1	All are zeros
CV_T.4 mm.1	All are zeros
Apex.2	All are zeros
Thinnest.4 mm.1	Constant value
LocationX.3	All are zeros
LocationY.3	All are zeros
CSI_T.2	All are zeros
SD_T.4 mm.2	All are zeros
CV_T.4 mm.2	All are zeros
RMS_E.4 mm.2	All are zeros
SR_E.4 mm.2	All are zeros
En.Anterior	Same string
ESI.Anterior	Not part of input data

Abbreviation: ESI, ectasia screening index

### 3.3 Target label encoding

**Figure 2:**
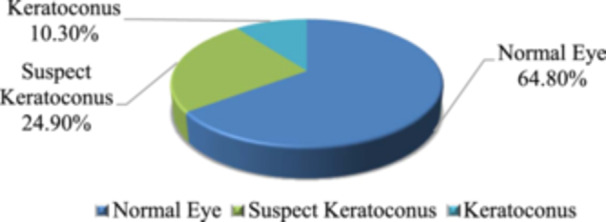
Existence of 3 different classes in the data set.

Since this is a classification problem, there is a target column which is in fact a range of values, called the ESI value, between 0 and 100 generated by the SS‐1000 CASIA OCT Imaging Methods. We have changed the target parameter into three integers, 0, 1, 2. Where 0 means normal eye, 1 means suspect keratoconus eye and 2 means keratoconus eye. Target label encoding is shown in Table 2. After encoding the target label, we got 10.3% keratoconus eye, 24.9% suspect keratoconus eye and 64.8% normal eye which is shown in Figure 2.

**Table 2:** Ectasia screening index.

ESI interval	Diagnosis	Classes
ESI ∊ [0 − 4]	Normal Eye	Class 0
ESI ∊ [5–29]	Suspect Keratoconus	Class 1
ESI ∊ [30 − 100]	Keratoconus	Class 2

Abbreviation: ESI, ectasia screening index.

### 3.4 Data standardization

Data standardization is the process of transforming data into a common format or scale to make it consistent and comparable. It involves techniques like mean‐centering, variance scaling, and categorical data encoding. Here, standard scaler is used, which normalizes features by subtracting the average and adjusting to have a standard deviation of one. The standard score of a sample x is calculated as:

(1) $$z=\frac{\left(x-u\right)}{s}$$

Where, u is the mean of the training samples, and s is the standard deviation of the training samples.

### 3.5 Feature ranking and selection

**Figure 3:**
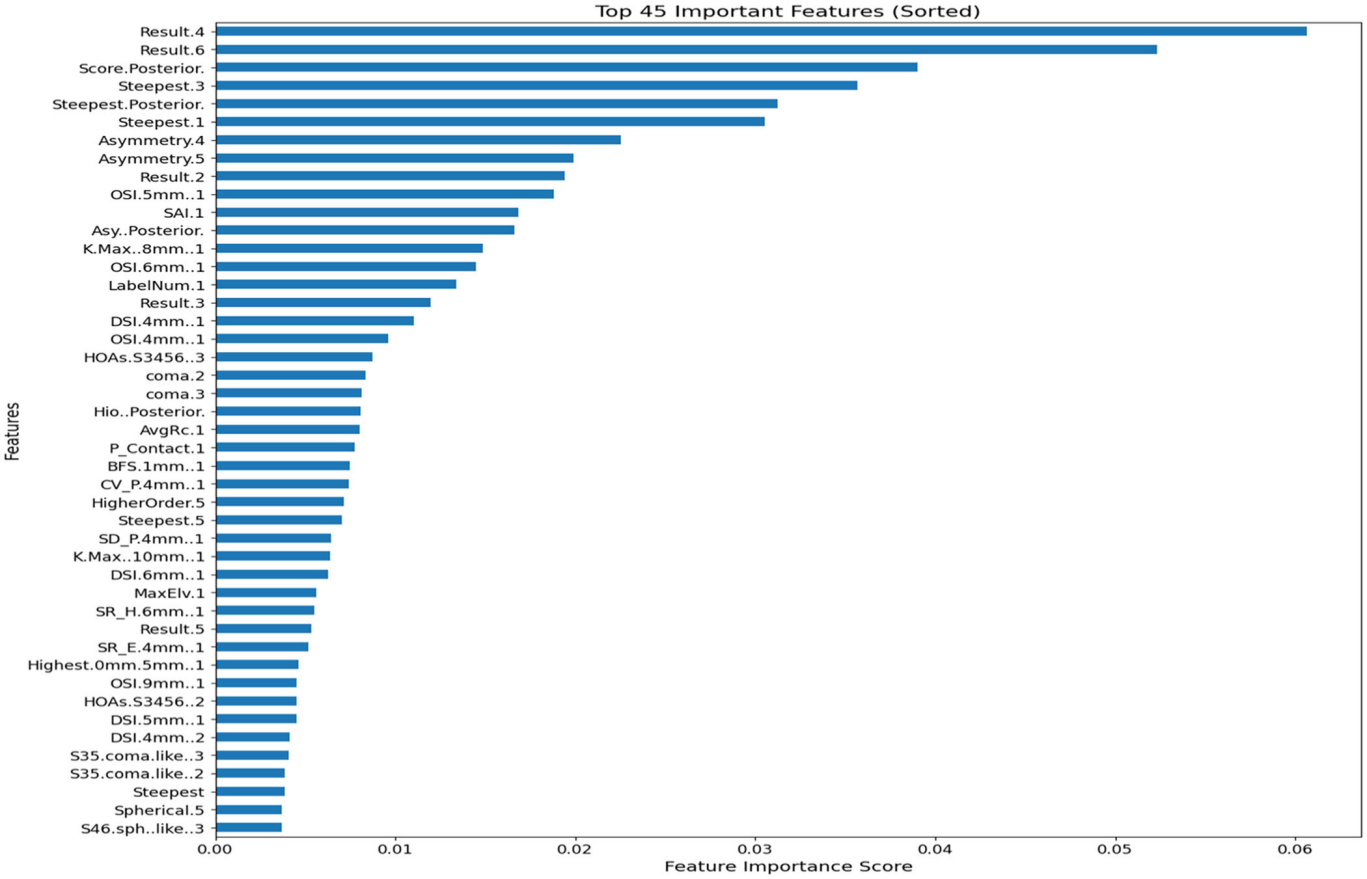
Important features with their corresponding scores prediction.

Feature ranking is the process of evaluating the importance of individual data features to determine their impact on the outcome of interest, helping with feature selection and improving data analysis or machine learning models. Here, the Extremely Randomized Trees Classifier (Extra Trees Classifier) is used for feature ranking. It is a kind of decision tree method that generates predictions using a collection of random trees. By randomly choosing a subset of the parameters and dividing the data according to the optimal split among these features, the technique creates several trees, commonly known as an Extra‐ Trees Forest. The average of all the forest's trees' projections is used to determine the outcome. The top 45 features are shown in Figure 3 with their corresponding scores for prediction.

## 4 BASIC MACHINE LEARNING MODEL

Numerous machine learning classifiers are used in various combinations to develop an accurate ensemble prediction model. We carefully analyzed each classifier's strengths and weaknesses. In our study, we evaluated the efficacy of four machine learning classifiers—SVM, DT, RF, and ANN—in predicting hypertension. The same data set was used to train each classifier. We conducted independent evaluations of each classifier. The classifier's performance was further investigated in various ensemble combinations. In addition, the effectiveness of various multi‐level stacked ensemble combinations was investigated. Each is given a brief description. The classifier employed in this study is shown below.

### 4.1 Support vector machine (SVM)

SVM is a classification technique that can be used in machine learning. SVM finds a hyperplane in a high‐dimensional space that divides the data into classes. SVM can be used to find three hyperplanes that split the data into three classes in a three‐class problem. There are various ways that can be used while working with a 3‐class problem using SVM, depending on the specific problem and data set. One popular strategy is to utilize a “one‐vs‐one” method, in which SVM is used to create a new classifier for each possible pair of classes. As a result, there are three classifiers in all. When predicting the class of a new data point, each classifier is applied to the data point, and the most commonly predicted class is chosen as the final prediction.

### 4.2 Decision tree (DT)

In a multi‐class classification problem, decision trees work by creating a tree‐like structure where each internal node represents a decision based on a specific feature, and each leaf node represents a class label. The algorithm recursively splits the data into smaller subsets based on the selected features until the subsets are pure or can no longer be further split. The selected features are ranked based on their information gain, which measures how much a feature contributes to reducing the impurity in the data.

### 4.3 Artificial neural network (ANN)

Our ANN is a feed‐forward neural network with three layers: an input layer, two hidden layers, and an output layer. The input layer has a number of nodes equal to the number of input features in the data set. The two hidden layers have 128 and 64 nodes, respectively, and use the rectified linear unit (ReLU) activation function. The output layer has three nodes, which correspond to the three possible classes in the classification problem, and uses the softmax activation function to predict class probabilities.

### 4.4 Random forest (RF)

We have a data set with three classes, and we've determined the top 15 features for predicting these classes. The RF technique would then construct numerous decision trees from a random selection of the characteristics and data. The method would feed each data point through each decision tree, and each tree would predict the class of the data point depending on the features it employed. The mode of the classes predicted by all decision trees would be the final forecast for that data point.

### 4.5 Ensemble techniques

The method we have proposed is an ensemble learning method that employs four different classifiers: SVM, DT, RF, and ANN. Herein, SVM is well‐suited for high‐dimensional data like the data set we used, known for good performance in classification tasks, and effective in handling small datasets and DT offers interpretability, allowing us to understand the decision‐making process, and can handle mixed data types (categorical and numerical). In addition, RF combines multiple decision trees, reducing overfitting and improving generalization, robust to outliers and noise. Moreover, ANN is powerful for complex, nonlinear relationships, potentially capturing underlying patterns in the data that other algorithms might miss. The goal is to improve prediction accuracy by combining the predictions of these individual classifiers. The method begins by importing the required libraries, which are pandas, numpy, and scikit‐learn (sklearn). The pandas library is used to manipulate and analyze data, while numpy is used for scientific computing. Machine learning algorithms for classification, regression, and clustering are available in the scikit‐learn library.

## 5 EVALUATION METRICS

In this research, we have a two‐fold objective: first, to classify Keratoconus, and second, to evaluate and measure the effectiveness and performance of the proposed approach by comparing it with existing methods. Table 3 displays the potential outcomes of a confusion matrix in 3‐class classification. True Positive (TP), False Positive (FP), and False Negative (FN) are the three possible outcomes.

**Table 3:** Confusion matrix for three‐class classification.

Actual label	Predicted label		
	Class 0	Class 1	Class 2
Class 0	TP	FN	FN
Class 1	FP	TP	FN
Class 2	FP	FP	TP

*Note*: FN: The number of instances classified incorrectly as not belonging to each class. FP: The number of instances classified incorrectly as belonging to each class when they do not.TP: The number of instances correctly classified as belonging to each of the three classes (Class 0, Class 1, and Class 2).

### 5.1 Accuracy

In a three‐class classification, accuracy is a measure of how many instances are classified correctly out of the total instances. The accuracy can be calculated using the following equation:

(2) $$\mathrm{Accuracy}=\frac{\mathrm{TP}1+\mathrm{TP}2+\mathrm{TP}3}{\mathrm{Total Instances}}$$

Where,

TP1: The number of instances classified correctly as belonging to Class 1.

TP2: The number of instances classified correctly as belonging to Class 2.

TP3: The number of instances classified correctly as belonging to Class 3.

Total Instances: The total number of instances in data set.

### 5.2 Precision

Precision is a performance metric that evaluates how well a model predicts the positive outcomes of a classification task, like three‐class classification. In the context of a three‐class classification problem, precision can be defined independently for every class. The formula for precision for a specific class (e.g., Class 1) is as follows:

(3) $$\mathrm{Precision}\;(\mathrm{Class}\;1)=\frac{\mathrm{TTP}\;(\mathrm{True Positives for Class}\;1)}{\mathrm{TP}\;(\mathrm{True Positives for Class}\;1)+\mathrm{FP}\;(\mathrm{False Positives for Class}\;1)}$$

Where,

True Positives (TP) for Class 1: The number of instances classified correctly as belonging to Class 1.

False Positives (FP) for Class 1: The quantity of cases that are incorrectly classified as Class 1 even though they are not Class 1.

### 5.3 Recall

Recall, also known as Sensitivity or True Positive Rate, is another significant performance metric used in classification tasks such as three‐class classification. The ability of a model to correctly identify all positive instances within a given class is measured by recall. In the context of a three‐class classification problem, recall can be defined independently for each class. The formula for recall for a specific class (e.g., Class 1) is as follows:

(4) $$\mathrm{Recall}\;(\mathrm{Class}\;1)=\frac{\mathrm{TP}\;(\mathrm{True Positives for Class}\;1)}{\mathrm{TP}\;(\mathrm{True Positives for Class}\;1)+\mathrm{FN}\;(\mathrm{False Negatives for Class}\;1)}$$

Where,

True Positives (TP) for Class 1: The number of cases correctly classified as Class 1.

False Negatives (FN) for Class 1: The number of cases incorrectly classified as not belonging to Class 1 when they do.

### 5.4 F1 score

The F1 score is a prominent performance metric in classification tasks such as three‐class classification. It combines precision and recall into a single metric, providing a balanced assessment of a model's ability to make accurate positive predictions while avoiding missing positive instances. The formula for the F1 score for a specific class (e.g., Class 1) is as follows:

(5) $$F1\;\mathrm{Score}\;(\mathrm{Class}\;1)=\frac{2\;*\;\mathrm{Precision}\;(\mathrm{Class}\;1)\;*\;\mathrm{Recall}\;(\mathrm{Class}\;1)}{\mathrm{Precision}\;(\mathrm{Class}\;1)+\mathrm{Recall}\;(\mathrm{Class}\;1)}$$

Where,

Precision (Class 1): As explained earlier, this measures the accuracy of positive predictions for Class 1.

Recall (Class 1): As explained earlier, this measures the model's ability to correctly identify all positive instances for Class 1.

## 6 EXPERIMENTAL SETUP

For data analysis, evaluation and testing our proposed model, we have taken the help of Python programming language with version 3.10. And all the analysis and evaluation have been done in Google Colaboratory online. The parameters used for training different model are listed in Table 4. The table contains the meaning and used value for each parameter.

**Table 4:** Different parameters of different models with their values to train them.

Model	Parameter	Description	Used value
SVM	kernel	Defines how data points are transformed for classification.	linear
	gamma	Controls the influence of individual training examples on the model.	auto
	C	Balances fitting the training data with avoiding overfitting.	2
	probability	Enables probability estimates for SVM classification outcomes.	True
	random_state	Ensures reproducibility of training results by controlling randomness.	0
DT	splitter	Defines the strategy for splitting nodes in the decision tree.	best
	max_depth	Sets the maximum complexity of the decision tree.	None
	random_state	Ensures reproducibility of training results by controlling randomness.	0
RF	n_estimators	Specifies the number of decision trees used in the ensemble model.	100
	random_state	Ensures reproducibility of training results by controlling randomness.	0
XGBoost	n_estimators	Specifies the number of decision trees used in the ensemble model.	100
	random_state	Ensures reproducibility of training results by controlling randomness.	0
	objective	Defines the optimization goal for XGBoost, such as minimizing classification error.	binary:logistic
AdaBoost	n_estimators	Specifies the number of decision trees used in the ensemble model.	100
	learning_rate	Determines the step size for updating weights of weak learners in AdaBoost.	1
	random_state	Ensures reproducibility of training results by controlling randomness.	0
LR	random_state	Ensures reproducibility of training results by controlling randomness.	0
ANN	Activation	Calculates the output of a node based on its inputs and their weights.	Relu, softmax
	layers	Defines the number of hidden layers in the artificial neural network.	3
	nodes	Sets the number of processing units within each layer of the ANN.	128,6,3
	Loss	Measures the difference between the model's predictions and actual outcomes.	Sparse_categorical_crossentropy
	Optimizer	Controls how the weights of the ANN are adjusted during training.	adam

Abbreviations: ANN, artificial neural network; DT, decision tree, RF, random forest; SVM, support vector machine.

## 7 RESULT ANALYSIS AND DISCUSSION

The evaluation of the keratoconus detection model's effectiveness relies on its metric scores. To enhance clarity, this study's overall results are divided into two main phases. Phase 1 concentrates on the performance attributes of the proposed keratoconus detection scheme, examining various feature sets both with and without data standardization. In Phase 2, a comparative analysis is conducted, evaluating the overall results across multiple individual machine learning classifiers, and contrasting them with state‐of‐the‐art models in the field of keratoconus detection.

### 7.1 Phase 1: Experimental results for different feature set with and without data standardization

The effectiveness of the Keratoconus Detection System (KDS) model is gauged by assessing its performance across a variety of metrics. A higher performance level is indicated by elevated values of metrics such as accuracy, precision, recall, F1‐Score as shown in Equations ((2), (3), (4), (5)) to demonstrate better detection capabilities. Table 5 in the study presents the experimental results of keratoconus detection. To facilitate meaningful comparative analysis, the results of individual metrics are compared for different feature sets with and without data standardization. This comparison allows for an assessment of how data set balancing impacts the model's performance across various evaluation metrics.

**Table 5:** Experimental results for different feature sets with and without data standardization.

Metrics		Class	Without data standardization			With data standardization		
			45 Feature	30 Feature	15 Feature	45 Feature	30 Feature	15 Feature
Accuracy (%)			98.20	98.50	**99.00**	98.00	98.90	**99.80**
Precision		Class 0	0.99	0.99	**0.99**	0.99	0.98	**1.00**
		Class 1	0.98	0.98	**0.99**	0.98	0.99	**0.99**
		Class 2	0.98	0.98	**1.00**	0.98	0.98	**1.00**
Recall		Class 0	0.98	0.98	**0.99**	0.98	0.98	**1.00**
		Class 1	0.99	**1.00**	0.99	0.99	0.99	**0.99**
		Class 2	0.98	0.98	**0.99**	0.99	0.97	**1.00**
F1‐ Score		Class 0	0.97	**1.00**	0.99	0.97	0.99	**1.00**
		Class 1	0.97	0.99	**0.99**	0.98	0.98	**0.99**
		Class 2	0.98	0.98	**0.99**	0.99	0.98	**1.00**

*Note*: Bold values indicate the best performances in terms of different performance metrics.

**Figure 4:**
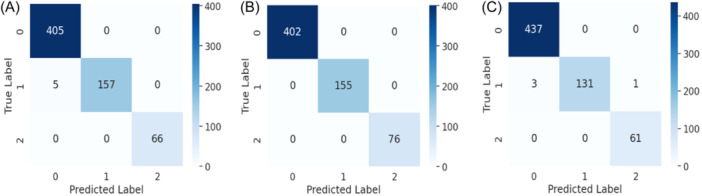
Confusion matrix for KeratoEL with data standardization for top (A) 45 features, (B) 30 features, & (C) 15 features.

**Figure 5:**
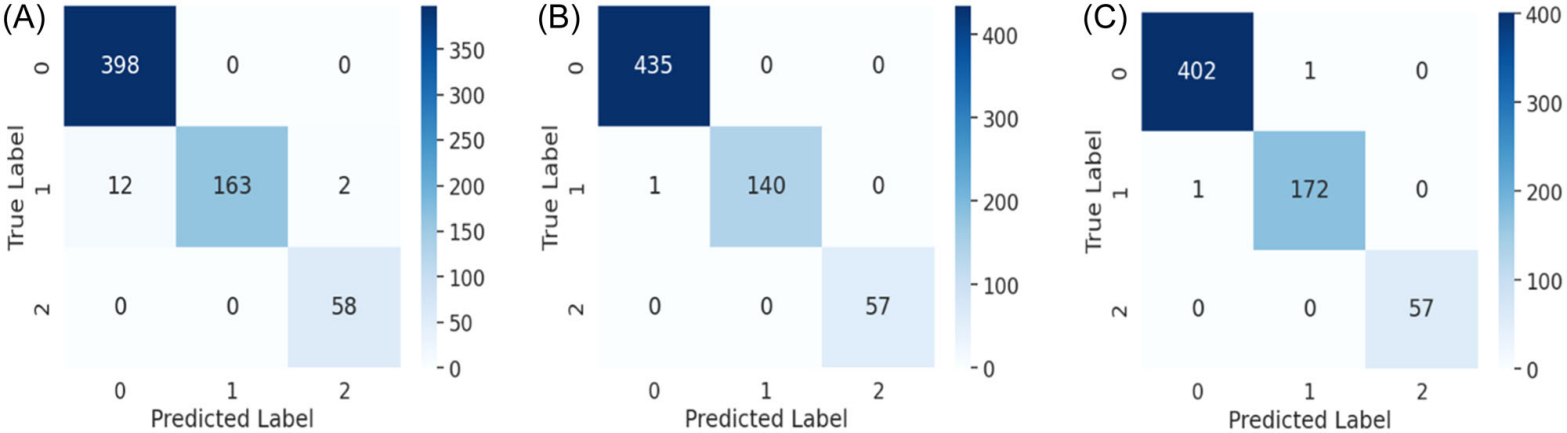
Confusion matrix for KeratoEL without data standardization for top (A) 45 features, (B) 30 features, & (C) 15 features.

In the field of machine learning, the primary objective of feature selection is to choose the most pertinent features to enhance model performance, mitigate overfitting, expedite computation, and improve interpretability. In our study, Feature Set 1 comprises top 45 features, while Feature Set 2 and Feature Set 3 consist of top 30 and 15 features, respectively, out of 448 features. From Table 5, it can be seen that for data standardization and without data standardization the evaluation performance remains nearly unchanged. But for smaller feature sets, the increment of performance is noticeable and significant. When the top 15 features are selected, the highest accuracy of 99.8% and 99% is obtained for data standardization and without data standardization respectively. These top 15 features are most significant compared to others which leads to improved performance. The corresponding confusion matrix that is obtained for different feature sets with and without data standardization is shown in Figures 4, 5. The confusion matrix reveals that the false positive value and false negative value are significantly lower, highlighting the superior performance characteristics of the proposed model.

**Figure 6:**
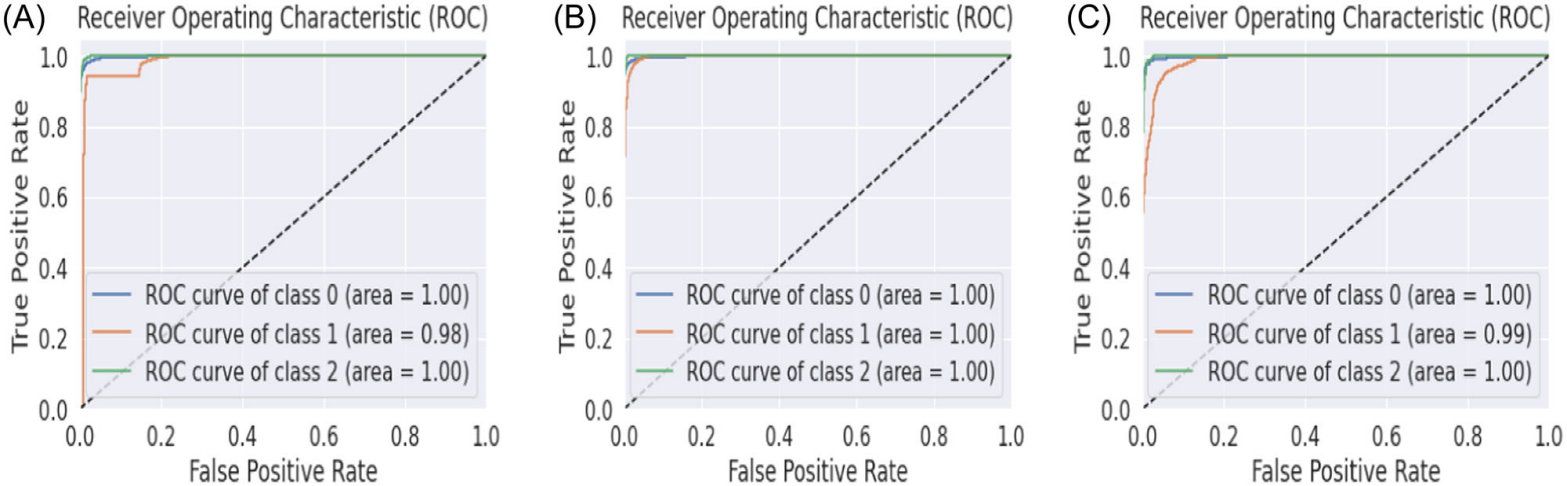
ROC curves for KeratoEL with data standardization for top (A) 45 features, (B) 30 features, & (C) 15 features.

**Figure 7:**
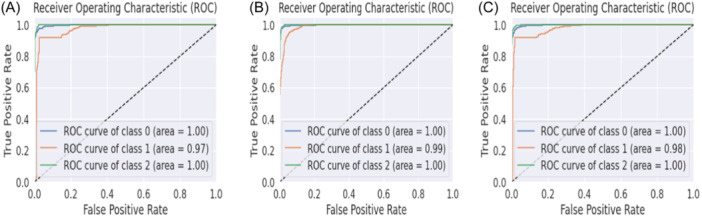
ROC curves for KeratoEL without data standardization for top (A) 45 features, (B) 30 features, & (C) 15 features.

ROC curve analysis is a graphical method used to assess the performance of classification models. It plots the trade‐off between true positive rate (sensitivity) and false positive rate (1 ‐ specificity) at different decision thresholds. A steeper ROC curve that's closer to the top‐left corner indicates a better model. The area under the ROC curve (AUC‐ROC) is a common performance metric, with a higher score indicating better performance. This analysis helps to select the appropriate threshold for classification tasks. The illustrations in Figures 6, 7 show the Area Under Curve (AUC) for the proposed KeratoEL model. The model has achieved perfect classification for classes 0 and 2, meaning that each occurrence of these classes has been accurately categorized, according to the AUC value of 1.00 for these classes. With only a few cases misclassified, the model exhibits nearly flawless classification accuracy for class 1, according to the AUC value of 0.99.

### 7.2 Phase 2: Comparative analysis between other methods for different feature set with and without data standardization

To demonstrate the effectiveness of the proposed KeratoEL model across a wider range of scenarios, we have overhauled the entire process for various traditional machine learning models and proposed model. The preprocessing steps throughout the workflow have largely remained the same, except for a key modification: instead of using the KeratoEL model, we employed a single classifier for keratoconus detection. We carried out a comprehensive comparative analysis on the data set, considering different feature sets, both with and without data standardization. The evaluation incorporates four critical metrics: accuracy, precision, recall, and F1‐score.

Tables 6, 7, 8 present the accuracy, precision, recall and F1‐score metrics for various classifiers with different number of features. The precision of the KeratoEL model varies between 0.98 and 1 for different features, whereas the lowest precision of 0.79 is achieved for the SVM algorithm without data standardization. Moreover, in the KeratoEL model, the recall metric ranges from 0.97 to 1 across various features, whereas AdaBoost attains its lowest recall score of 0.70 when applied to standardized data. In addition, the F1‐score ranges from 0.97 to 1 in the KeratoEL model, while AdaBoost achieves its lowest F1‐score of 0.79, whether the data is standardized or not.

**Table 6:** Evaluation metrics analysis of different classifiers for the top 45 features without and with data standardization.

Classifiers	Class	Without data standardization				With data standardization			
		Accuracy	Precision	Recall	F1‐score	Accuracy	Precision	Recall	F1‐score
SVM	0	89.70%	0.90	0.91	0.91	93.70%	0.92	0.95	0.94
	1		**0.79**	0.85	0.87		0.79	0.76	0.89
	2		0.92	0.85	0.82		0.95	0.81	0.88
DT	0	92.50%	0.88	0.93	0.92	93.50%	0.90	0.95	0.91
	1		0.95	0.90	0.88		0.87	0.90	0.87
	2		0.93	0.93	0.93		0.93	0.93	0.94
RF	0	91.90%	0.89	0.95	0.89	92.10%	0.93	0.95	0.94
	1		0.88	0.88	0.92		0.92	0.88	0.92
	2		0.92	0.92	0.92		0.91	0.92	0.96
XGBoost	0	94.50%	0.95	0.94	0.95	96.10%	0.96	0.97	0.95
	1		0.98	0.92	0.95		0.98	0.90	0.97
	2		0.88	0.89	0.87		0.88	0.90	0.87
AdaBoost	0	87.00%	0.81	0.95	0.92	89.60%	0.87	0.99	0.92
	1		0.88	0.71	**0.79**		0.82	**0.70**	**0.79**
	2		0.95	0.85	0.84		0.96	0.84	0.81
LR	0	88.60%	0.91	0.85	0.83	91.50%	0.94	0.95	0.93
	1		0.96	0.87	0.91		0.86	0.87	0.90
	2		0.91	0.83	0.84		0.91	0.83	0.83
ANN	0	96.00%	0.97	0.95	0.96	96.80%	0.98	0.96	0.97
	1		0.92	0.94	0.92		0.97	0.97	0.96
	2		0.97	0.95	0.92		0.96	0.95	0.97
KeratoEL	0	**98.20%**	**0.99**	**0.98**	**0.97**	**98.00%**	**0.99**	**0.98**	**0.97**
	1		**0.98**	**0.99**	**0.97**		**0.98**	**0.99**	**0.98**
	2		**0.98**	**0.98**	**0.98**		**0.98**	**0.99**	**0.99**

Abbreviations: ANN, artificial neural network; DT, decision tree, RF, random forest; SVM, support vector machine

**Table 7:** Evaluation metrics analysis of different classifiers for the top 30 features without and with data standardization.

Classifiers	Class	Without data standardization				With data standardization			
		Accuracy	Precision	Recall	F1‐score	Accuracy	Precision	Recall	F1‐score
SVM	0	92.10%	0.93	0.95	0.97	95.20%	0.92	0.95	0.97
	1		0.86	0.77	0.87		0.86	0.76	0.89
	2		0.94	0.86	0.92		0.95	0.85	0.92
DT	0	93.50%	0.96	0.95	0.90	96.50%	0.97	0.95	0.91
	1		0.88	0.91	0.91		0.88	0.90	0.90
	2		0.92	0.94	0.94		0.95	0.93	0.94
RF	0	94.00%	0.90	0.92	0.93	95.10%	0.93	0.94	0.96
	1		0.95	0.88	0.92		0.94	0.89	0.92
	2		0.92	0.93	0.96		0.91	0.92	0.96
XGBoost	0	95.00%	0.97	0.97	0.94	97.50%	0.98	0.97	0.95
	1		0.96	0.95	0.97		0.98	0.96	0.96
	2		0.91	0.90	0.93		0.95	0.90	0.94
AdaBoost	0	87.30%	0.91	0.90	0.91	91.30%	0.91	0.95	0.92
	1		0.83	0.80	**0.79**		0.82	0.78	**0.79**
	2		0.92	0.90	0.91		0.96	0.84	0.91
LR	0	90.10%	0.96	0.93	0.93	93.10%	0.95	0.95	0.93
	1		0.91	0.89	0.90		0.91	0.88	0.90
	2		0.92	0.87	0.88		0.91	0.88	0.89
ANN	0	97.00%	0.97	0.95	0.97	97.30%	0.98	0.95	0.98
	1		0.96	0.96	0.96		0.95	0.96	0.96
	2		0.95	0.95	0.96		0.93	0.99	0.98
KeratoEL	0	**98.50%**	**0.99**	**0.98**	**1.00**	**98.90%**	**0.98**	**0.98**	**0.99**
	1		**0.98**	**1.00**	**0.99**		**0.99**	**0.99**	**0.98**
	2		**0.98**	**0.98**	**0.98**		**0.98**	**0.97**	**0.98**

*Note*: Bold values indicate best and worst cases.

Abbreviations: ANN, artificial neural network; DT, decision tree, RF, random forest; SVM, support vector machine

**Table 8:** Evaluation metrics analysis of different classifiers for the top 15 features without and with data standardization.

Classifiers	Class	Without data standardization				With data standardization			
		Accuracy	Precision	Recall	F1‐score	Accuracy	Precision	Recall	F1‐score
SVM	0	94.50%	0.96	0.98	0.97	96.80%	0.97	0.99	0.98
	1		0.92	0.92	0.92		0.93	0.92	0.93
	2		0.97	0.95	0.95		0.98	0.92	0.95
DT	0	93.80%	0.95	0.93	0.94	97.50%	0.98	0.99	0.98
	1		0.96	0.95	0.96		0.97	0.98	0.97
	2		0.97	0.92	0.92		0.98	0.98	0.98
RF	0	94.50%	0.96	0.97	0.97	98.10%	0.97	0.99	0.98
	1		0.94	0.94	0.94		0.98	0.98	0.98
	2		0.97	0.97	0.95		0.99	0.98	0.97
XGBoost	0	95.60%	0.96	0.97	0.97	98.50%	0.94	0.99	0.97
	1		0.94	0.95	0.96		0.99	0.99	0.98
	2		0.96	0.95	0.96		0.98	0.98	0.98
AdaBoost	0	89.60%	0.95	0.98	0.96	94.00%	0.96	0.98	0.97
	1		0.91	0.81	0.86		0.94	0.81	0.87
	2		0.86	0.85	0.94		0.94	0.92	0.93
LR	0	92.50%	0.88	0.96	0.93	95.00%	0.96	0.98	0.97
	1		0.93	0.89	0.91		0.92	0.88	0.90
	2		0.92	0.93	0.92		0.97	0.93	0.95
ANN	0	98.00%	0.99	0.98	0.98	98.73%	0.99	0.98	0.98
	1		0.97	0.97	0.97		0.97	0.98	0.97
	2		0.99	0.97	0.98		1.00	0.97	0.98
KeratoEL	0	**99.00%**	**0.99**	**0.99**	**0.99**	**99.80%**	**1.00**	**1.00**	**1.00**
	1		**0.99**	**0.99**	**0.99**		**0.99**	**0.99**	**0.99**
	2		**1.00**	**0.99**	**0.99**		**1.00**	**1.00**	**1.00**

Abbreviations: ANN, artificial neural network; DT, decision tree, RF, random forest; SVM, support vector machine

**Figure 8:**
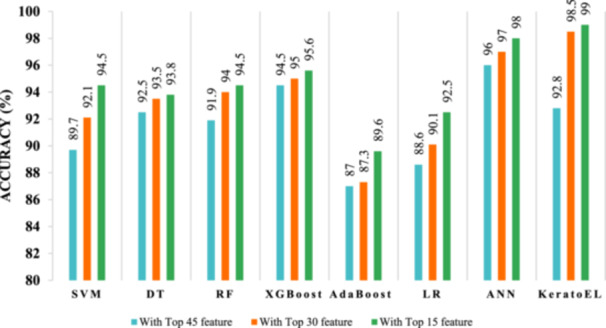
Comparison graph of different classifiers for three different feature groups (45, 30, and 15) with their corresponding accuracy score (without standardization). ANN, artificial neural network; DT, decision tree, RF, random forest; SVM, support vector machine

**Figure 9:**
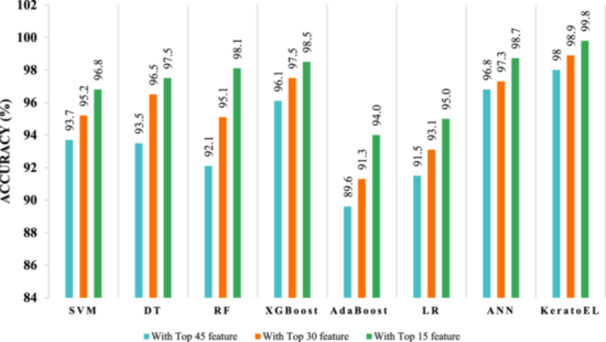
Comparison graph of different classifiers for 3 different feature groups (45, 30, and 15) with their corresponding accuracy score (with standardization). ANN, artificial neural network; DT, decision tree, RF, random forest; SVM, support vector machine

Figures 8, 9 depict the accuracy of different classifiers for different features in the data set with and without data standardization. Figure 8 shows that the proposed KeratoEL model is able to achieve the highest accuracy of 99% with 15 features, whereas the AdaBoost algorithm, utilizing the top 45 features, attains the lowest accuracy of 87% without data standardization. Moreover, the KeratoEL model with the top 15 features has the highest accuracy of 99.8%, while the AdaBoost algorithm with the top 45 features has the lowest accuracy of 89.6% for standardized data, as shown in Figure 9. We can a get little bit of improved performance if we standardize the data. Furthermore, reducing the number of features can improve the performance of any model. From the above analysis, it is clear that the proposed KeratoEL model is more effective and efficient than a single ML model.

Table 9 shows the comparison of the proposed Ensemble model with state‐of‐the‐art models in terms of accuracy and AUC. The performance of the proposed keratoEL for three class problems (e.g., Normal Eye, Suspect Keratoconus, and Keratoconus) is compared with previous studies. The authors of^6^, ^20^ have used a similar data set for this work, where they have utilized t‐SNE and RF methods and obtained accuracy of 94.10% and 94% respectively. The highest accuracy and AUC of KeartoEL are 99.8% and 0.99 respectively, which are better than previous works, whether the data set is similar or different. This comparison shows the superiority of the proposed KeratoEL over previous studies.

**Table 9:** Comparison with state‐of‐the‐art models.

Paper	Imaging Device	Number of Classes	Method	Accuracy (%)	AUC
[17]	Scheimpflug tomographer	5	SVM	98.9	0.99
[6]	SS‐1000 CASIA OCT	3	PCA, t‐SNE	94.10	‐
[18]	Auto‐Keratometer	2	Logistic Regression	86.7	‐
[5]	SS‐1000 CASIA OCT	3	SVM	94.00	0.95
[20]	Placido disc analyzer	3	RF	91.50	0.97
[21]	Scheimpflug tomographer	2	CNN	99.50	0.99
KeratoEL	SS‐1000 CASIA OCT	3	Ensemble	99.80	0.99

Abbreviations: CNN, convolutional neural network; DT, decision tree, RF, random forest; SVM, support vector machine

## 8 CONCLUSIONS

The primary benefit of the proposed model lies in its seamless integration into the diagnostic process. This work's primary contribution is the development of an ensemble ML model, namely KeratoEL, to aid ophthalmologists in keratoconus disease detection. ML algorithms hold the promise of revolutionizing traditional medical screening programs, offering rapid diagnostics and improving patient care and comfort in the process. Herein, the corneal tomography image is used as the input of the proposed model, which then evaluates whether the individual is affected by keratoconus or suspect keratoconus or non‐keratoconus. The proposed KeratoEL model achieves an accuracy range of 98%–99.8% for different feature sets. From the obtained results, it is evident that the proposed KeratoEL model ensures a notably high level of performance. To summarize, this paper introduces the development of a screening tool utilizing a ML algorithm to autonomously identify keratoconus disease by analyzing corneal tomography. The proposed KeratoEL model can be integrated into the device used for tomography, serving as an additional feature to aid ophthalmologists in efficiently screening their patients. In the future, this ML model will help to facilitate the detection of keratoconus and reduce the corneal transplant cases. In the future, the proposed model will be employed for early stage keratoconus detection using various new datasets to verify its applicability in a wider range.

## AUTHOR CONTRIBUTIONS

**Prodeep Kumar Paul**: Conceptualization; methodology; software; writing—original draft; validation. **Arif Hossan**: Supervision; formal analysis; writing—review ānd editing. **Shah Muhammad A. Ullah**: Methodology; writing—review ānd editing; investigation; validation.

## CONFLICT OF INTEREST STATEMENT

The authors declare no conflict of interest.

## ETHICS STATEMENT

No human subjects were involved in this research. We have used real life cornea parameters of human subjects from well‐known online database namely, Harvard Dataverse.^22^ Therefore, we do not require patient consent.

## TRANSPARENCY STATEMENT

The lead author Arif Hossan affirms that this manuscript is an honest, accurate, and transparent account of the study being reported; that no important aspects of the study have been omitted; and that any discrepancies from the study as planned (and, if relevant, registered) have been explained.

## Data Availability

Data will be provided upon request. The code is available at http://github.com/arifkuet/Keratoconus-Detection
